# Nitrogen Oxyanion-dependent Dissociation of a Two-component Complex That Regulates Bacterial Nitrate Assimilation*

**DOI:** 10.1074/jbc.M113.459032

**Published:** 2013-09-04

**Authors:** Victor M. Luque-Almagro, Verity J. Lyall, Stuart J. Ferguson, M. Dolores Roldán, David J. Richardson, Andrew J. Gates

**Affiliations:** From the ‡ Centre for Molecular and Structural Biochemistry and; § School of Biological Sciences, University of East Anglia, Norwich NR4 7TJ, United Kingdom,; the ¶ Departamento de Bioquímica y Biología Molecular, Campus de Rabanales, Universidad de Córdoba, Córdoba 14071, Spain, and; the ‖ Department of Biochemistry, University of Oxford, Oxford OX1 3QU, United Kingdom

**Keywords:** Bacterial Metabolism, Bacterial Signal Transduction, Ligand-binding Protein, Nitrogen Metabolism, Protein-Protein Interactions, Nitrate Assimilation, RNA Signaling, Two-component Regulator

## Abstract

Nitrogen is an essential nutrient for growth and is readily available to microbes in many environments in the form of ammonium and nitrate. Both ions are of environmental significance due to sustained use of inorganic fertilizers on agricultural soils. Diverse species of bacteria that have an assimilatory nitrate/nitrite reductase system (NAS) can use nitrate or nitrite as the sole nitrogen source for growth when ammonium is limited. In *Paracoccus denitrificans*, the pathway-specific two-component regulator for NAS expression is encoded by the *nasT* and *nasS* genes. Here, we show that the putative RNA-binding protein NasT is a positive regulator essential for expression of the *nas* gene cluster (*i.e. nasABGHC*). By contrast, a nitrogen oxyanion-binding sensor (NasS) is required for nitrate/nitrite-responsive control of *nas* gene expression. The NasS and NasT proteins co-purify as a stable heterotetrameric regulatory complex, NasS-NasT. This protein-protein interaction is sensitive to nitrate and nitrite, which cause dissociation of the NasS-NasT complex into monomeric NasS and an oligomeric form of NasT. NasT has been shown to bind the leader RNA for *nasA*. Thus, upon liberation from the complex, the positive regulator NasT is free to up-regulate *nas* gene expression.

## Teaser

**Background:** Nitrogen oxyanion-responsive two-component regulators that control assimilatory nitrate metabolism in heterotrophic bacteria are poorly characterized.

**Results:** The *nasT* and *nasS* genes encode a regulatory complex that dissociates upon sensing nitrate/nitrite, releasing the RNA-binding protein NasT.

**Conclusion:** NasS-NasT is a two-component regulator for nitrate/nitrite perception.

**Significance:** A nitrate/nitrite-sensitive interaction between NasS and NasT has been demonstrated for the first time.

## Introduction

A supply of bioavailable nitrogen can be a limiting factor for the growth of bacteria in both terrestrial and aquatic environments. Although these organisms readily assimilate inorganic nitrogen from ammonium (NH_4_^+^), many species have been shown to use nitrate (NO_3_^−^) or nitrite (NO_2_^−^) as their sole source of nitrogen. The ability to assimilate these readily water-soluble oxyanions is particularly widespread in heterotrophic bacteria and is associated with the expression of a cytoplasmic assimilatory NO_3_^−^/NO_2_^−^ reductase system (NAS)^4^ that performs the two-electron reduction of NO_3_^−^ to NO_2_^−^, followed by the six-electron reduction of NO_2_^−^ to NH_4_^+^ (1–5). The NH_4_^+^ formed from the NO_3_^−^ assimilation pathway can fuel reactions that yield l-glutamate, which plays a pivotal role in biosynthetic cellular metabolism. For example, under nitrogen-sufficient growth conditions, NH_4_^+^ may be used directly via a reaction with 2-oxoglutarate that is mediated by glutamate dehydrogenase. Alternatively, when the availability of NH_4_^+^ is limited, the bulk of l-glutamate is formed by the concerted action of the NH_4_^+^-dependent glutamine synthetase and the glutamine:2-oxoglutarate amidotransferase (also known as glutamate synthase) in the glutamine synthetase/glutamine:2-oxoglutarate amidotransferase cycle (6).

*Paracoccus denitrificans* PD1222 has recently been shown to assimilate inorganic nitrogen from NO_3_^−^ or NO_2_^−^ via an NADH-dependent NAS system encoded by the *nasABGHC* genes (hereafter termed the *nas* gene cluster) (5). NAS activity could be clearly measured in cytoplasmic extracts prepared from cells grown with NO_3_^−^ as the sole nitrogen source. However, activity was not detected in extracts prepared from cells grown in NH_4_^+^-sufficient culture medium (5). This is consistent with other studies involving Gram-negative bacteria, in which expression of the NO_3_^−^/NO_2_^−^ assimilation pathway is subject to tight hierarchical control involving (i) primary induction by the general nitrogen regulatory system during NH_4_^+^ starvation (7) and (ii) additional system-specific NO_3_^−^/NO_2_^−^-responsive regulatory proteins typically encoded within, or in close proximity to, *nas* loci (2, 8–10).

Pathway-specific control of bacterial NO_3_^−^ assimilation has been extensively studied in *Klebsiella pneumoniae* M5al. Here, the key regulator NasR is an example of a single-component NO_3_^−^/NO_2_^−^-responsive transcription antiterminator protein for which the signal transduction mechanism has been studied in detail (8). NasR polypeptides comprise an N-terminal NO_3_^−^/NO_2_^−^-sensing NIT domain fused to a C-terminal ANTAR (AmiR and NasR transcription antitermination regulator) signaling domain. This arrangement has been recently confirmed by structural resolution of the NasR protein, which exists as a homodimer in the absence of inducer, *i.e.* the “inactive” state (11). The regulatory target for the NasR ANTAR signaling domain is a *cis*-acting regulatory element or “antiterminator” secondary structure within the leader region of the *nasFEDCBA* mRNA transcript (12, 13).

In addition to NasR, a two-component regulatory system (NasS-NasT) has been proposed to be involved in the specific control of NO_3_^−^ assimilation in the diazotrophs *Azotobacter vinelandii* (2) and *Rhodobacter capsulatus* (4) and in members of the *Pseudomonas* genus such as *Pseudomonas aeruginosa* (10) and *Pseudomonas putida* JLR11 (14). Bioinformatics analyses of bacterial genome sequences suggest that *nasT* and *nasS* are widely distributed in Gram-negative bacteria that assimilate NO_3_^−^ and NO_2_^−^, including important symbionts, pathogens, and denitrifiers (5, 9, 10, 15). In *P. denitrificans*, a putative NO_3_^−^-sensing two-component regulatory system is encoded by the *nasT* and *nasS* genes, which are located immediately upstream of the *nas* gene cluster on chromosome II (5, 9).

NasT is a member of the ANTAR protein family (15). In contrast to NasR, NasT does not contain any recognized NO_3_^−^/NO_2_^−^-sensing domain. Instead, NasS belongs to the small molecule-binding protein superfamily, the members of which are typically present in ABC-type transport systems. One such example includes the cyanobacterial NO_3_^−^-binding protein NrtA from *Synechocystis* sp. PCC 6803, which has been structurally characterized, revealing a single NO_3_^−^ anion bound at a defined site within the protein (16). In *A. vinelandii*, the phenotypes of *nasS* and *nasT* strains suggest that NasS and NasT proteins play negative and positive regulatory roles in assimilatory NO_3_^−^/NO_2_^−^ reductase gene expression, respectively (2). This is consistent with NasS and the ANTAR-type protein NasT being a two-component configuration for regulation of *nas* gene expression in which the sensor and signal transduction functions are segregated into different proteins, *i.e.* NasS and NasT, respectively. However, to our knowledge, neither a protein-protein interaction between NasS and NasT nor direct NO_3_^−^/NO_2_^−^ sensing by NasS has yet been experimentally demonstrated. In this work, focused on the *P. denitrificans* NAS pathway, we present the first biochemical characterization of a NO_3_^−^/NO_2_^−^-responsive two-component system (NasS-NasT), in which binding of NO_3_^−^ or NO_2_^−^ by the sensor NasS triggers release of the positive RNA-binding regulator NasT.

## EXPERIMENTAL PROCEDURES

### 

#### 

##### Bacterial Strains, Media, and Growth Conditions

*P. denitrificans* PD1222 was routinely cultured under aerobic conditions at 30 °C in either LB medium or a defined mineral salts medium (5) supplemented with ammonium chloride (10 mm), potassium nitrate (20 mm), potassium nitrite (10 mm), or sodium l-glutamate (5 mm) as the sole nitrogen source as required. *Escherichia coli* strains were cultured aerobically in LB medium at 37 °C unless stated otherwise. Cell growth was followed by measuring the absorbance of cultures at 600 nm (*A*_600_). Antibiotics were used at the indicated final concentrations: ampicillin, 100 μg/ml; gentamycin, 20 μg/ml; kanamycin, 25 μg/ml; rifampicin, 100 μg/ml; spectinomycin, 25 μg/ml; and streptomycin, 60 μg/ml.

##### Construction of nasT and nasS Strains

*P. denitrificans* mutant strains were constructed by replacement of significant portions of the target gene essentially as described previously (5). To generate the *nasT* strain (*nasT*Δ::streptomycin), the front and rear sections of the *nasT* gene were amplified from genomic DNA isolated from *P. denitrificans* PD1222 in separate reactions using oligonucleotide primer sets T1/T2 and T3/T4 (Table 1), respectively. Reactions were performed using the Expand High Fidelity PCR system (Roche Applied Science). A BamHI restriction site was introduced into the end of each fragment, allowing ordered assembly of the gene sections within the multiple cloning site of the pGEM-T Easy vector (Promega). The resulting construct had a unique BamHI site at the interface of the front and rear sections of *nasT*, into which a streptomycin resistance cassette, obtained from pSRA2, was introduced (17). The *nasT*Δ::streptomycin fragment was then transferred to the mobilizable vector pSUP202* (5) as an EcoRI fragment. The *nasS* strain (*nasS*Δ::kanamycin) was constructed in a similar manner. PCR amplification of the front and rear gene sections was performed using primer sets S1/S2 and S3/S4 (Table 1), respectively, and the fragments were then cloned into pGEM-T Easy. A kanamycin resistance cassette derived from pSUP2021 was inserted into a unique BamHI site between the front and rear sections of *nasS*. The *nasS*Δ::kanamycin fragment was transferred to the mobilizable vector pSUP202* as an EcoRI fragment. All cloning steps were carried out using an *E. coli* DH5α host following standard transformation and ligation protocols (18). Conjugation, selection, and validation of mutants were performed as described previously (5).

**TABLE 1:** **Primers used in this study**

Primer	Sequence (5′ → 3′)
T1 (forward)	AACGGAATTCGCATCCAGCAACCCCCTGATT
T2 (reverse)	ATCGCGGATCCAAGGCGCTTGTCCATCTGCT*^a^*
T3 (forward)	ATCGCGGATCCGGATATCGGCCTATGTC*^a^*
T4 (reverse)	TACGCGTCGACCGTCGAGAAATGGAAC
S1 (forward)	TACGGAATTCACATCGTGCTGATCGACCTG
S2 (reverse)	ATCGCGGATCCAAATCATGGCCCTGTTC*^a^*
S3 (forward)	ATCGCGGATCCCGAATATCTGGACCTG*^a^*
S4 (forward)	ACGCGTCGACCTTTCGGAGGAGAGGATTT
TS1 (forward)	CACGCTAGCAGAGGATCGCATCACCATCACCATCACGGATCCATCGAGGGAAG*^b^*
	GGACAGGCGCCTTTCGATCGTCGTCATC
TS2 (reverse)	GCAAAGCTTTCAGCCGGCGAAGGGTGGTTCGAAG*^c^*
SA1 (forward)	TAATACGACTCACTATAGGGGAACCACCCTTCGCCGGCTGAGCGTTTTGCAGGCA*^d^*
SA2 (reverse)	AACGGCGGGCATGGTGGCTCCGATGCGTT
AB1 (forward)	TAATACGACTCACTATAGGACCGGGCTGGTCGAGCAGGTGATGAA*^d^*
AB2 (reverse)	GGTGCCGTCCTTCTGGATATTGGCGTGGTT
SDH1 (forward)	TAATACGACTCACTATAGGCGATCCTGCACACGCTCTATGGCCAGTCGC*^d^*
SDH2 (reverse)	AGATGCCGGTCGGGTGGAACTGCACGAACT

*^a^* The BamHI site is underlined.

*^b^* The NheI is underlined.

*^c^* The HindIII is underlined.

*^d^* The T7 promoter is underlined.

##### Assay for Assimilatory NO_3_^−^/NO_2_^−^ Reductase Activity in P. denitrificans Strains

NADH-dependent assimilatory NO_3_^−^/NO_2_^−^ reductase activity was measured in cytoplasmic extracts as described previously (5). Given that NADH is consumed at a ratio of ∼3:1 NO_2_^−^:NO_3_^−^, NAS activity assay was performed with NO_2_^−^ as the electron acceptor to allow rapid reproducible initial rate determinations using cytoplasmic extracts prepared from relatively small cell volumes.

##### Cloning, Expression, and Purification of NasT and NasS

A 1.75-kb fragment containing the coding regions for *nasT* and *nasS* was amplified by PCR. Reactions containing 5% (v/v) Me_2_SO were performed essentially as described by Sambrook and Russell (18) using primers TS1 and TS2 (Table 1). The purified product was cloned into pGEM-T Easy and then transferred to the pET-24a expression vector (Novagen) as an NheI-HindIII restriction fragment. The resulting construct, pET-24a/*nasTS*, was sequenced and transformed into *E. coli* BL21(DE3) for protein expression. Cells containing the expression plasmid were grown at 37 °C in 500 ml of LB medium until cultures reached an *A*_600_ reading of ∼0.5. Expression was induced by addition of 1 mm isopropyl β-d-thiogalactopyranoside, after which the culture temperature was lowered to 28 °C.

Cells were harvested 3 h after induction by centrifugation at 12,000 × *g* for 20 min at 4 °C. Soluble cell extracts were prepared at 4 °C. Pellets were resuspended in buffer A (20 mm sodium phosphate, 150 mm NaCl, 25 mm imidazole (Sigma-Aldrich), and 10% (v/v) glycerol, pH 7.0). The cell suspension was supplemented with a protease inhibitor mixture (cOmplete, EDTA-free, Roche Applied Science) and 1 mg/ml lysozyme (hen egg white, EC 3.2.1.17, Fluka) and incubated at 4 °C for 30 min. Cell lysis was achieved following addition of 1% (v/v) Triton X-100 (Sigma-Aldrich) to the mixture and incubation on a rocking platform for 10 min. The lysate was supplemented with DNase I (bovine pancreas, EC 3.1.21.1, Sigma) and RNase A (bovine pancreas, EC 3.1.27.5, Sigma) and then incubated for a further 10 min, after which it was fluid. Insoluble cell debris was removed by ultracentrifugation at 260,000 × *g* for 60 min at 4 °C.

NasS-NasT was purified by immobilized metal affinity chromatography (IMAC), followed by anion-exchange and size-exclusion chromatography. All purification steps were performed at 4 °C with a flow rate of 1 ml/min unless stated otherwise. Soluble cell extract prepared from a 2-liter culture was loaded onto a 10-ml Ni^2+^ IMAC column (HiTrap Chelating HP, GE Healthcare) that was precharged with nickel sulfate, washed with analytical reagent-grade water, and equilibrated with buffer A. Following loading, the column was then washed with a further 4 column volumes of buffer A to elute unbound protein. Bound protein was eluted with a linear gradient of 25–500 mm imidazole applied over 5 column volumes. Fractions containing NasS and NasT were pooled and buffer-exchanged into buffer B (50 mm NaHEPES, 1 mm EDTA, and 10% (v/v) glycerol, pH 7.0). This sample was loaded onto a 5-ml HiTrap Q HP anion-exchange column (GE Healthcare) that was pre-equilibrated with buffer B. Elution of bound protein was achieved by applying a linear gradient of 0–2 m NaCl over five column volumes. Peak fractions containing NasS-NasT were then pooled, buffer-exchanged into buffer C (50 mm NaHEPES and 100 mm NaCl, pH 7.0), and concentrated by ultrafiltration. Samples were loaded onto a 70-ml preparative size-exclusion column (Sephacryl S-200 high resolution, GE Healthcare) that was pre-equilibrated with buffer C. Protein concentration was determined by bicinchoninic acid assay (19).

For identification of purified proteins, bands corresponding to the correct molecular mass of NasS (∼42 kDa) and NasT (∼22 kDa) were excised from denaturing SDS-polyacrylamide gels. Each gel slice was washed, reduced, alkylated, and treated with trypsin according to standard procedures adapted from Shevchenko *et al.* (20). The tryptic peptide fragments were analyzed by mass spectrometry using an ultraflex^TM^ MALDI-TOF/TOF spectrometer (Bruker). Briefly, 0.5–0.8 μl of the peptide samples was applied to a Prespotted AnchorChip^TM^ MALDI target plate (Bruker), and the spots were washed with 10–15 μl of 10 mm ammonium phosphate and 0.1% trifluoroacetic acid according to the manufacturer's protocol. The instrument was then calibrated using the prespotted standards. Samples were analyzed using a flexControl^TM^ method (version 3.0, Bruker) optimized for peptide detection. Acquired spectra were processed using flexAnalysis^TM^ (version 3.0, Bruker). The resulting peak lists were used for a database search using an in-house Mascot® 2.4 server (Matrix Science, London, United Kingdom). The search was performed on the UniProt Swiss-Prot/TrEMBL database (release 20121031) with taxonomy set to bacteria and on a common contaminants database using the trypsin/P enzyme with a maximum of one missed cleavage, a peptide mass tolerance of 50 ppm, carbamidomethylation as fixed, and oxidation and acetylation (protein N terminus) as variable modifications. Using those parameters, Mascot protein scores >85 were significant (*p* < 0.05). NasS and NasT peptides were identified with significance scores of 187 (sequence coverage of 45%, expect value of 6.7 × 10^−13^) and 123 (sequence coverage of 65%, expect value of 1.79 × 10^−6^), respectively.

##### UV-visible Electronic Absorbance and Fluorescence Spectroscopy

Absorbance spectra were recorded for purified protein (∼1.5 mg/ml) using a Hitachi U-3000 spectrophotometer. An extinction coefficient of 47,100 m^−1^ cm^−1^ at 280 nm was estimated for the NasS-NasT complex. Emission spectra were recorded at 295 nm using a Varian Cary Eclipse fluorescence spectrophotometer. Curve fitting was performed using Origin 7.0 (OriginLab Corp.).

##### Analytical Ultracentrifugation and Size-exclusion Chromatography

Analytical ultracentrifugation sedimentation equilibrium experiments were performed at 20 °C using a Beckman Optima XL-I analytical ultracentrifuge equipped with an integrated UV-visible absorbance optical system and an An-50 Ti analytical rotor (Beckman Instruments). Protein samples and buffer controls were loaded into the relevant sectors of two-channel EPON cells (1.2-cm path). Sedimentation equilibrium profiles were recorded at 280 nm at a range of protein concentrations (3–23 μm) and rotation speeds (7.5, 12, and 16 krpm). The partial specific volume for NasS-NasT was calculated as 0.744 ml/g using the sedimentation interpretation program SEDNTERP (version 20120111 BETA, Biomolecular Interactions Technology Centre). Analytical ultracentrifugation experiments were performed according to published methods (21). Data analysis was performed using the UltraScan II software package (version 9.9) (22). Fitting of sedimentation profiles to an ideal one-component model was used to determine the apparent molecular masses of proteins.

Analytical size-exclusion chromatography was performed on a 24-ml Superdex 200 HR 10/30 column (Amersham Biosciences) that was equilibrated with buffer C. The column was loaded with 250 μl of purified protein (1.5 mg/ml) and developed at a flow rate of 0.5 ml/min. Time elution of protein from the column was followed automatically at 280 nm using an ÄKTA fast protein liquid chromatograph (GE Healthcare). The low aromatic amino acid content of NasT did, however, complicate assigning the retention peak position at 280 nm. Instead, this was done manually by measuring the absorbance of column fractions at 260 nm. The apparent molecular masses of NasS-NasT and the isolated NasS and NasT proteins were estimated by comparison with known protein standards supplied in a gel-filtration calibration kit (high molecular weight kit, GE Healthcare), which were run individually under the relevant buffer conditions.

##### Protein-RNA Binding Monitored by Electrophoretic Mobility Shift Assay

RNA molecules for the *nasA* leader and regions of *nasB* and *sdhA* were prepared by *in vitro* transcription. Primer sets SA1/SA2, AB1/AB2, and SDH1/SDH2 (Table 1) were used in separate PCRs to generate DNA (∼300 bp) for the putative control region upstream of the *nasA* gene and regions of *nasB* and *sdhA* genes as controls. The T7 promoter sequence was included in forward primers SA1, AB1, and SDH1 for subsequent RNA transcription. The *in vitro* synthesis of single-stranded RNA was performed with the HiScribe^TM^ T7 *in vitro* transcription kit (New England Biolabs). Overnight reactions were performed at 42 °C. DNA template was then removed by DNase treatment at 37 °C for 30 min. RNA products were visualized on 1.5% agarose gels stained with ethidium bromide.

For electrophoretic mobility shift assays, ∼20 μm purified NasS-NasT was incubated with different concentrations of RNA (50–90 nm) for 15 min at room temperature in 10-μl binding reaction volumes. The buffer contained 10 mm Tris, pH 7.4, 150 mm KCl, 0.1 mm EDTA, 0.1 mm dithiothreitol, and 1 mm NaNO_3_. Samples were loaded onto native 5% polyacrylamide gels (37.5:1 acrylamide:bisacrylamide) in 45 mm Tris, 45 mm boric acid, and 1 mm EDTA, pH 8.3. Gels were stained for RNA using a SYBR® Green EMSA kit (Molecular Probes) and visualized in an FX scanner (Bio-Rad). After electrophoresis, the shifted band present in *lane 4* of Fig. 3*A* was excised and prepared for MALDI-TOF-MS analysis for protein identification as described above. The NasT peptide was identified with a significance score of 87.

## RESULTS

### 

#### 

##### Role of NasT and NasS in NO_3_^−^/NO_2_^−^ Assimilation

**FIGURE 1.**
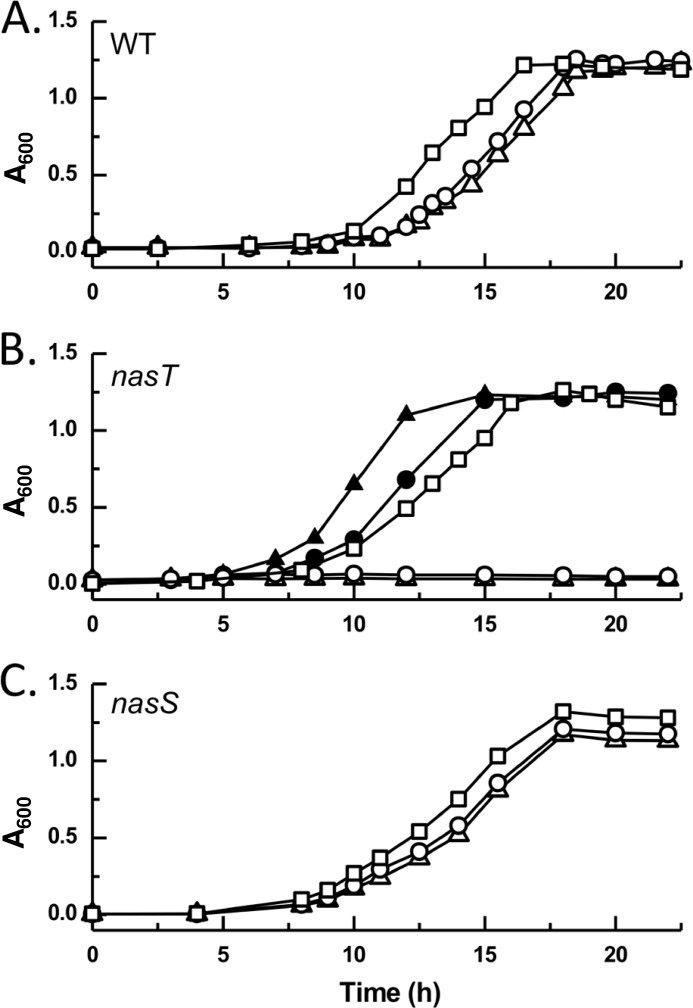
**Aerobic growth of *P. denitrificans* strains on different nitrogen sources.** Growth curves are shown for WT (*A*), *nasT* (*B*), and *nasS* (*C*) strains with NH_4_^+^ (□), NO_3_^−^ (○), or NO_2_^−^ (▵) present as the sole nitrogen source in minimal salts medium. Growth curves for the *nasT* strain complemented with the pEG276-*nasT* expression plasmid are shown in *B* during growth on NO_3_^−^ (●) or NO_2_^−^ (▴). The results shown are the average of triplicate determinations.

In the absence of NH_4_^+^, *P. denitrificans* may use NO_3_^−^ or NO_2_^−^ as the sole nitrogen source for growth (Fig. 1*A*), an ability that has been directly linked to expression of the *nas* gene cluster (5). To explore the role of the NasS-NasT two-component regulator in expression of the NAS system, *P. denitrificans* strains that were deficient in either *nasT* or *nasS* were constructed. A *nasT* strain was unable to grow with either NO_3_^−^ or NO_2_^−^ as the sole nitrogen source, but growth of this strain was unaffected when cells were grown in the presence of NH_4_^+^ (Fig. 1*B*) or l-glutamate (data not shown). Growth on NO_3_^−^ and NO_2_^−^ could be restored to WT levels upon complementation with a functional gene copy that was expressed in *trans* from the expression vector pEG276-*nasT*.

NAS expression is induced in WT cells by NO_3_^−^ when NH_4_^+^ is absent and can be monitored by assaying NADH-dependent NO_2_^−^ reductase activity in cytoplasmic extracts (hereafter termed NAS activity). The level of enzyme activity detected in induced WT cells (14.8 ± 1.2 units) was similar to that described previously (5). However, this NAS activity was not detectable in cytoplasmic extracts prepared from the *nasT* strain grown on l-glutamate with or without the additional inclusion of NO_3_^−^ (Table 2). NAS activity was restored to WT levels in the *nasT* strain when the deletion was complemented with a functional plasmid-borne gene copy. This is consistent with NasT being a positive regulator of the NO_3_^−^/NO_2_^−^ assimilation pathway.

**TABLE 2:** **Analysis of NAS expression in *P. denitrificans* WT, *nasT*, and *nasS* strains** NADH-dependent NO_2_^−^ reductase activity was measured in cytoplasmic extracts prepared from cells grown in minimal medium containing l-glutamate that was supplemented additionally with NO_3_^−^ as inducer for expression of the NAS system. Activity was measured in nmol/min/mg of protein. ND, not detectable.

Strain	Growth conditions	
	l-Glutamate	l-Glutamate + NO_3_^−^
WT	<0.5	14.8 ± 1.2
*nasT*	ND	ND
*nasT*/pEG276-*nasT*		20.5 ± 0.8
*nasS*	13.5 ± 2.7	24.6 ± 0.5

Using NO_3_^−^ or NO_2_^−^ as the nitrogen source, a strain in which the *nasS* gene was mutated showed no clear growth defect with respect to the WT (Fig. 1*C*). However, in contrast to the WT, NAS activity could be readily detected in cytoplasmic extracts prepared from cells grown on l-glutamate (13.5 ± 2.7 units) despite omission of NO_3_^−^ as inducer for *nas* expression. The level of NAS activity present in the *nasS* strain was comparable to that observed in NO_3_^−^-induced WT cells (Table 2). That disruption of *nasS* did not have any pronounced effect on growth but instead led to the deregulation of NAS activity (such that it became constitutive irrespective of the presence of NO_3_^−^) is consistent with NasS normally having an inhibitory role in expression of the *nas* gene cluster.

##### Coexpression and Purification of NasS-NasT

**FIGURE 2.**
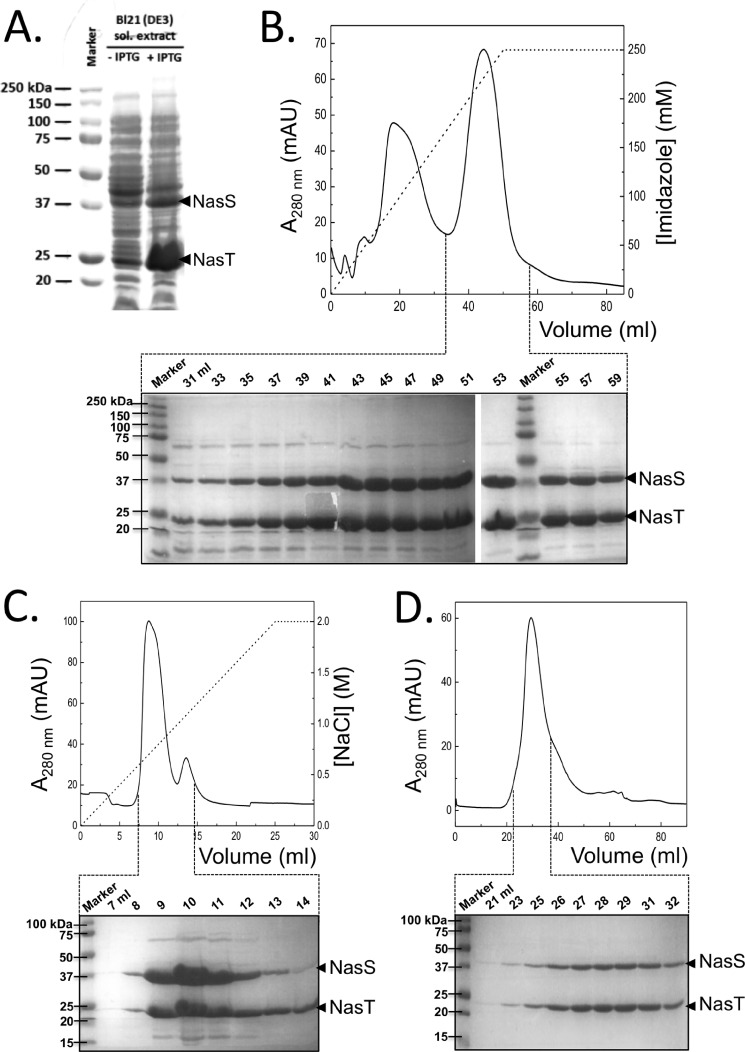
**Expression and co-purification of NasS and NasT.** Shown are the results from overexpression of recombinant *P. denitrificans* NasS and NasT proteins in *E. coli* BL21(DE3) (*A*), Ni^2+^ IMAC affinity purification of the NasS-NasT complex from the soluble (*sol.*) cell extract (*B*), and further purification of the NasS-NasT complex by anion-exchange (*C*) and size-exclusion (*D*) chromatography. Protein expression and purification were assessed by SDS-PAGE using Coomassie Brilliant Blue staining. *IPTG*, isopropyl β-d-thiogalactopyranoside; *mAU*, milli-absorbance units.

The mechanism by which the putative NO_3_^−^ sensor NasS and the ANTAR-type protein NasT cooperate to control *nas* gene expression in bacteria is unclear but may involve a protein-protein interaction (2). To investigate whether NasS and NasT interact, the expression construct pET-24a/*nasTS* was produced, which would not only yield high levels of recombinant forms of *P. denitrificans* NasS and NasT proteins but would also provide a means of immobilizing NasT via a polyhistidine tag during affinity purification. SDS-PAGE analysis of soluble protein extracts prepared from *E. coli* host cells containing the pET-24a/*nasTS* construct showed clear overexpression of two proteins at ∼22 and 42 kDa when isopropyl β-d-thiogalactopyranoside was added to the cell cultures (Fig. 2*A*).

Soluble extracts containing NasS and NasT were subjected to Ni^2+^ IMAC, which revealed that both proteins bound tightly to the affinity matrix despite only NasT being His-tagged (Fig. 2*B*). At this stage, protein bands were extracted from SDS-polyacrylamide gels and identified by mass spectrometry, which confirmed the 22- and 42-kDa bands as the NasT and NasS proteins from *P. denitrificans*, respectively. Co-purification of NasS and NasT was also observed during the subsequent polishing steps of the purification, which included Q-Sepharose anion-exchange (Fig. 2*C*) followed by size-exclusion (Fig. 2*D*) chromatography, consistent with a strong protein-protein interaction.

##### Nitrate/Nitrite Binding and Dissociation of the NasS-NasT Complex

**FIGURE 3.**
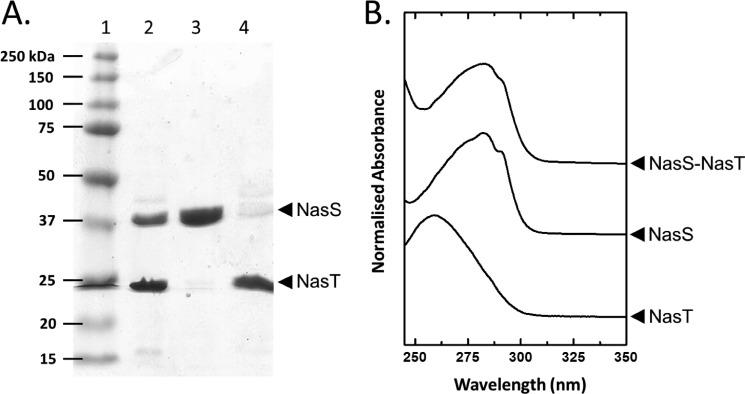
**NO_3_^−^-dependent dissociation of NasS from the immobilized NasS-NasT complex.** Shown are the results from SDS-PAGE analysis (*A*) and UV-visible electronic absorption spectroscopy (*B*) of the co-purified NasS-NasT and isolated NasS and NasT proteins. Purified NasS-NasT (*A*, *lane 2*) was bound to a Ni^2+^ IMAC column. NasS (*A*, *lane 3*) and NasT (*lane 4*) proteins were then obtained by sequential washing with buffer A supplemented with 1 mm NO_3_^−^ and 250 mm imidazole, respectively. Protein elution was assessed by SDS-PAGE using Coomassie Brilliant Blue staining. *Lane 1* contains molecular mass markers.

Co-purification of approximately equivalent amounts of NasS and NasT (as assessed visually by SDS-PAGE) was found to be dependent on the concentration of NO_3_^−^ present in solution. To explore the impact of NO_3_^−^ further, the purified NasS-NasT complex was re-immobilized on an IMAC column pre-equilibrated with buffer containing 50 mm NaHEPES and 100 mm NaCl, pH 7.5, that was additionally supplemented with 1 mm NO_3_^−^ as indicated (Fig. 3). In the absence of NO_3_^−^, NasS and NasT readily co-eluted upon washing the column with 500 mm imidazole (Fig. 3*A*, *lane 2*). In stark contrast, nearly complete separate elution of NasS was observed when a column containing freshly immobilized NasS-NasT was washed with binding buffer containing NO_3_^−^ (Fig. 3*A*, *lane 3*). Step elution of the remaining bound protein, the overwhelming majority being His-tagged NasT, was achieved by washing the column with imidazole (Fig. 3*A*, *lane 4*). Dissociation of NasS from the bound NasS-NasT complex was also observed in similar experiments in which NO_2_^−^ was used in place of NO_3_^−^, but dissociation was minimal in buffers supplemented with sulfate (SO_4_^2−^) (data not shown).

Inspection of the NasS polypeptide sequence reveals that this putative NO_3_^−^ sensor contains seven tryptophan residues. Accordingly, a clear shoulder at ∼288 nm was present in the UV-visible electronic absorbance spectrum of the purified NasS-NasT complex and the isolated NasS protein (Fig. 3*B*). By contrast, NasT contains no tryptophan residues. This is consistent with the relatively weak absorbance at 288 nm in the UV-visible spectrum of the isolated NasT protein (Fig. 3*B*).

**FIGURE 4.**
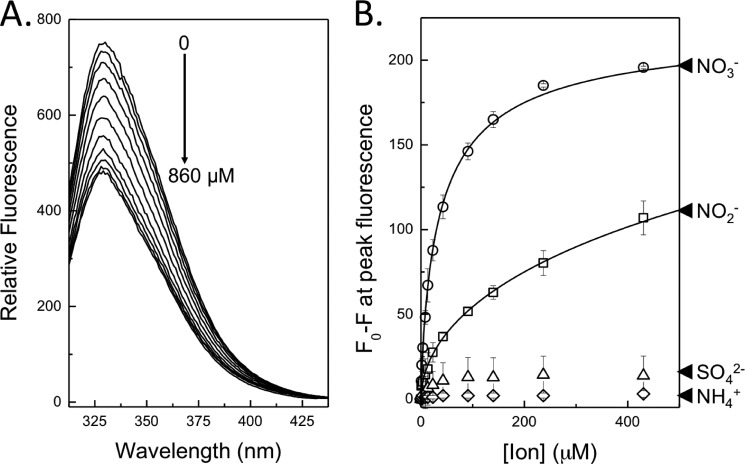
**Ligand binding properties of NasS-NasT as reported by the intrinsic tryptophan fluorescence of the NasS protein.** *A*, fluorescence quench of the tryptophan emission peak in response to increasing concentrations of NO_3_^−^. *B*, the effect of NaNO_3_ (○), NaNO_2_ (□), Na_2_SO_4_ (▵), and NH_4_Cl (♢) on peak fluorescence measured at 334 nm as a function of the solution concentration of the relevant ion. The excitation wavelength was 295 nm for the 1 μm NasS-NasT sample in 50 mm NaHEPES and 100 mm NaCl, pH 7.5.

The fluorescence emission spectrum of NasS-NasT that resulted from excitation at 295 nm revealed a clear peak at ∼334 nm, consistent with fluorescence of buried tryptophan residues (Fig. 4*A*). The magnitude of peak fluorescence was sensitive to NO_3_^−^ and was “quenched” to a resting value of ∼35% at concentrations above ∼250 μm. NasS-NasT fluorescence was also quenched by NO_2_^−^, indicating binding, but was insensitive to SO_4_^2−^ and NH_4_^+^ (Fig. 4*B*) and a range of other ionic compounds, including chloride (Cl^−^), chlorate (ClO_3_^−^), azide (N_3_^−^), and bicarbonate (HCO_3_^−^). That the other ions tested had little effect on the intrinsic protein fluorescence of NasS implies that the small molecule-binding site present in the NasS-NasT regulatory complex is specific to NO_3_^−^ but may also accommodate the smaller chemically similar anion NO_2_^−^.

The change in protein fluorescence (Δ*F*) observed in response to the solution concentration of ligand (L) can be explained by the following minimal binding model: NasS-NasT + L ↔ NasS-L + NasT. Here, the apparent equilibrium constant (*K*_*D*_^app^) for ligand binding and dissociation of the NasS-NasT complex was obtained by fitting the relevant data presented in Fig. 4*B* to the following equation: Δ*F* = [L]/(*K*_*D*_^app^ + [L]). *K*_*D*_^app^ values of 15 ± 2 and 94 ± 12 μm were determined with NO_3_^−^ and NO_2_^−^, respectively.

##### Solution State Properties of the NasS-NasT Complex

**FIGURE 5.**
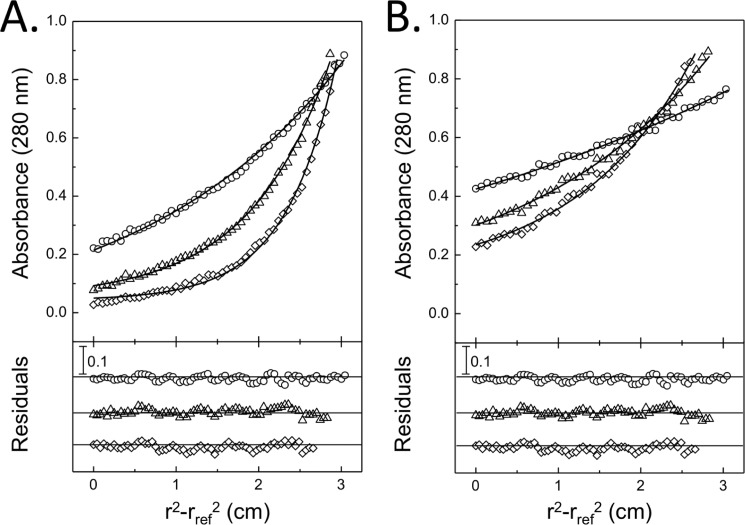
**Analytical ultracentrifugation sedimentation equilibrium profiles of the purified NasS-NasT complex.** *A* and *B*, sedimentation profiles in the absence and presence of 1 mm NO_3_^−^, respectively. The buffer contained 50 mm NaHEPES and 100 mm NaCl, pH 7.5, and rotation speeds of 7.5 (○), 12 (▵), and 16 (♢) krpm were applied to 10 μm protein samples at 21 °C.

To establish the composition of the NasS-NasT complex in solution, analytical ultracentrifugation experiments and size-exclusion chromatography were performed. Fig. 5 shows the sedimentation profile of the purified NasS-NasT complex. In the absence of NO_3_^−^, the sedimentation equilibrium profile of this complex fitted well to a single component with an apparent molecular mass of 132 ± 5 kDa (Fig. 5*A*). Given that equivalent amounts of NasS and NasT were observed by SDS-PAGE, the experimental value determined is consistent with the expected value of 128 kDa for a heterotetrameric solution state complex consisting of two NasS proteins and two NasT proteins. Similar experiments performed in the presence of 1 mm NO_3_^−^ resulted in a decrease in the apparent molecular mass for the complex (Fig. 5*B*). Given the low aromatic residue content of NasT in comparison with NasS, the imbalance of extinction coefficients precludes accurate discrimination of the proteins in the analytical ultracentrifugation experiment. Therefore, the ∼2-fold decrease in the observed apparent molecular mass for NasS-NasT in the presence of NO_3_^−^ is qualitative but consistent with NO_3_^−^-mediated dissociation of the larger NasS-NasT complex to lower apparent molecular mass species.

**FIGURE 6.**
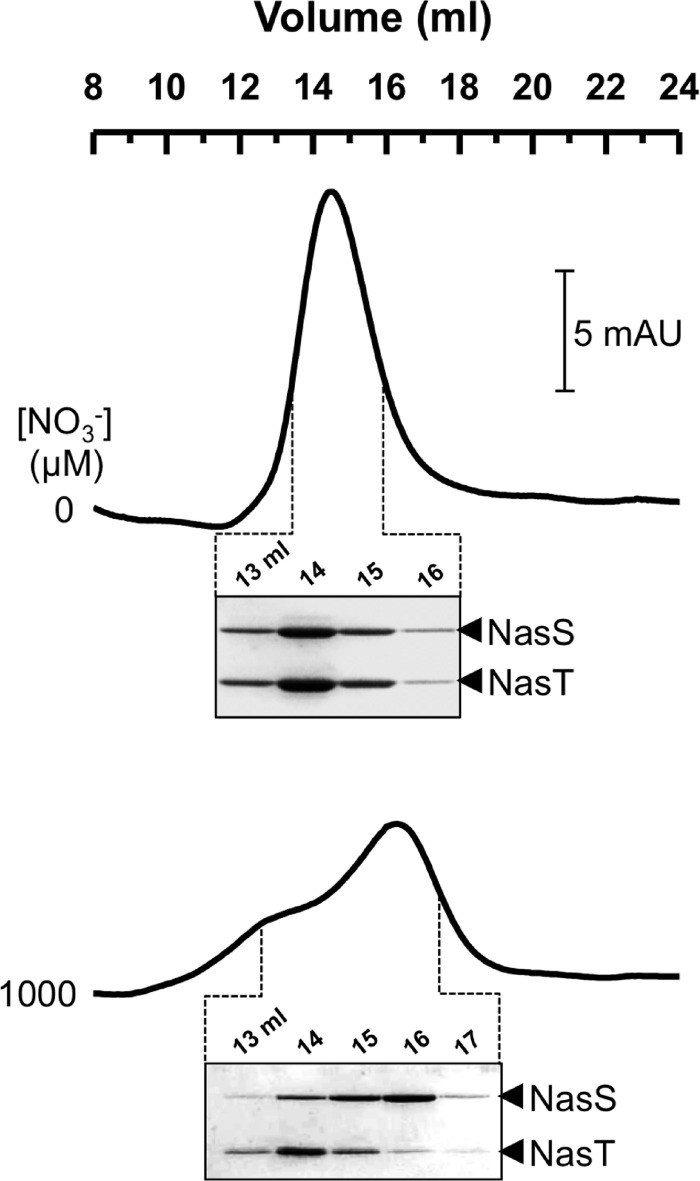
**Size-exclusion chromatography analysis of NO_3_^−^-dependent dissociation of purified NasS-NasT.** Representative chromatograms for protein elution recorded in the absence (*upper trace*) and presence (*lower trace*) of 1 mm NO_3_^−^ are shown. SDS-PAGE analysis of protein content for selected column fractions is shown below each chromatogram. Protein bands were visualized by Coomassie Brilliant Blue staining. *mAU*, milli-absorbance units.

Additional experiments involving analytical size-exclusion chromatography were performed to investigate the result of NO_3_^−^-dependent dissociation of the NasS-NasT complex in more detail (Fig. 6). In the absence of NO_3_^−^, NasS-NasT eluted at ∼14 ml as a single symmetrical peak. SDS-PAGE analysis revealed equivalent amounts of NasS and NasT in all fractions across this peak. An apparent molecular mass of 134 ± 10 kDa could be assigned to NasS-NasT by comparison with various protein standards applied to the same column under identical conditions. When the column equilibration buffer was supplemented with NO_3_^−^, an asymmetric protein elution profile was observed that was clearly different from that observed for the protein in the absence of NO_3_^−^.

SDS-PAGE analysis of eluted protein revealed differential elution of NasS and NasT. Peak NasS elution was observed at ∼16 ml, corresponding to an apparent molecular mass of 38 ± 5 kDa, consistent with a monomeric solution state for NasS. By contrast, the bulk of NasT eluted at ∼14 ml, corresponding to an apparent molecular mass of ∼130 kDa. Given that retention of NasT was within experimental error of that observed for the NasS-NasT complex prior to NO_3_^−^ exposure, the solution state of the isolated protein is considerably larger than would be expected for monomeric NasT (22 kDa) and implies that a substantial population of NasT can form a homo-oligomeric solution state when separated from NasS. Such behavior was observed at a range of pH values and salt concentrations and persisted despite the inclusion of the reducing agent dithiothreitol at 2 mm in all purification buffers. Thus, the multimeric state of NasT would likely consist of approximately six monomers and is unlikely to be the result of an adventitious protein-protein interaction. In summary, the combination of analytical ultracentrifugation and gel-filtration data provides compelling evidence that not only do NasS and NasT dissociate in the presence of NO_3_^−^, but that once separated, the NasT protein may also form a homo-oligomeric state in solution.

##### Specific Interaction of NasT with the Leader RNA of the nasA Gene

Analysis of the region upstream of *nasA* revealed repeated inverted sequence tracts for a series of regulatory hairpins similar to those present in the leader RNA of genes regulated by related RNA-binding proteins of the ANTAR signaling family (data not shown). The capacity of the NasS-NasT regulatory proteins to bind the leader RNA of the *nasA* gene from *P. denitrificans* was assessed in a series of electrophoretic mobility shift assays. Here, the purified NasS-NasT complex was “activated” by addition of 1 mm NO_3_^−^ and then incubated with RNA molecules produced by *in vitro* transcription. The RNAs tested included the predicted leader region of the *nasA* gene and two control sequences that included regions of the *nasB* and the *sdhA* genes, also from *P. denitrificans*, which did not contain similar hairpin structures associated with transcription antitermination.

**FIGURE 7.**
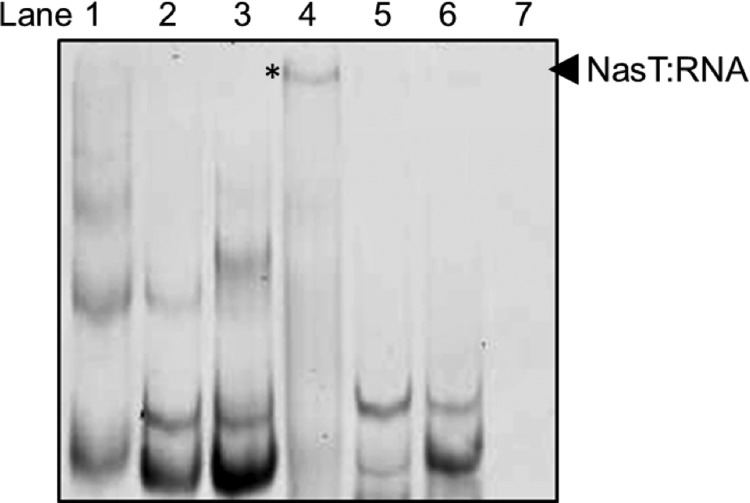
**Interaction of NasT with the leader RNA of *nasA*.** Approximately 70 nm *nasA* leader (*lane 1*), *nasB* (*lane 2*), and *sdhA* (*lane 3*) RNAs (each ∼300 nucleotides in length) were subjected to electrophoretic mobility shift assay. Similar experiments were performed with *nasA* (*lane 4*), *nasB* (*lane 5*), and *sdhA* (*lane 6*) RNAs that were preincubated with 20 μm purified NasS-NasT in the presence of 1 mm NaNO_3_ prior to loading. *Lane 7* was loaded with a control incubation of the NasS-NasT protein in binding buffer without RNA. RNA was resolved on native polyacrylamide gels and visualized using SYBR Green stain. The *asterisk* denotes the shifted band excised for identification by mass spectrometry.

The results presented in Fig. 7 reveal that the migration of the *nasA* RNA was significantly slower in the presence of the activated NasS-NasT protein relative to the migration of the same RNA when the protein was absent (compare *lanes 1* and *4*). In contrast, migration of either the *nasB* (Fig. 7, compare *lanes 2* and *5*) or *sdhA* (compare *lanes 3* and *6*) RNA molecules was essentially unaltered upon addition of activated NasS-NasT. When RNA was absent, the NasS and NasT proteins were not resolved by the staining procedure (Fig. 7, *lane 7*). Given that protein-nucleic acid complexes migrate more slowly than free linear nucleic acid fragments, the “mobility shift” observed for the leader RNA of the *nasA* gene in the presence of activated NasS-NasT is indicative of a specific protein-RNA interaction. The gel band in *lane 4* of Fig. 7 (denoted by an *asterisk*) was excised, and the protein component was identified as *P. denitrificans* NasT by mass spectrometry. This confirmed that the ANTAR protein NasT was responsible for RNA binding.

## DISCUSSION

Transcription antitermination is a control mechanism for gene expression that regulates a growing number of systems in bacteria, including those responsible for nitrogen metabolism (13, 23, 24). Specifically, one- and two-component systems (NasR and NasS-NasT, respectively) have been shown to regulate NO_3_^−^ assimilation. However, despite wide distribution among bacterial heterotrophs that assimilate NO_3_^−^ and/or NO_2_^−^, the biochemical properties of NasS-NasT two-component systems have been scarcely explored (2, 10, 14).

In this study, we have demonstrated that *nasT* is essential for growth of *P. denitrificans* with NO_3_^−^ or NO_2_^−^ as the sole nitrogen source. Deletion of *nasT* removes the capacity for NO_3_^−^/NO_2_^−^ induction of NAS expression. NasT polypeptides are predicted to contain an N-terminal CheY-like receiver domain (termed the REC domain) in addition to the C-terminal ANTAR domain similar to that present in NasR (26). The REC domain is found in a range of prokaryotic proteins that undergo distinctive conformational modulation during signal transduction as a consequence of covalent (*e.g.* phosphorylation) and/or physical (*e.g.* protein-protein interaction) modification by their cognate sensors (26, 27).

In this case, NasS is the cognate sensor. Sequence analysis reveals that NasS shares ∼44% sequence similarity with the cyanobacterial periplasmic NO_3_^−^-binding protein NrtA (16). Notably, NasS conserves all residues required for NO_3_^−^ coordination but lacks the N-terminal signal sequence and transmembrane helix present in NrtA required for periplasmic export and membrane localization, respectively (data not shown) (16). Accordingly, NasS is predicted to be a soluble cytoplasmic NO_3_^−^-binding protein. Given the subtlety of the distinguishing sequence features between NasS and related periplasmic NO_3_^−^-binding proteins, the *nasS* regulatory gene may have been incorrectly annotated in a number of bacterial genomes. Thus, the importance of the NasS-NasT system may have been underestimated.

Without *nasS*, *P. denitrificans* is unable to perceive the presence of the inducer (either NO_3_^−^ or NO_2_^−^), which results in the deregulation of gene expression such that the NAS system is expressed constitutively. That loss of NasS does not appear to significantly attenuate growth on NO_3_^−^ or NO_2_^−^ but instead leaves the bacterium unable to regulate expression of the NAS system suggests that NasS plays an inhibitory regulatory role in NAS expression when the inducer is absent. These results are consistent with the published phenotypes of *nasT* and *nasS* mutants in other bacteria (2, 10, 14) and imply that both NasS and NasT act together as a NO_3_^−^/NO_2_^−^-responsive two-component regulatory system to control *nas* gene expression.

To transmit the induction signal from NasS to NasT, a putative regulatory interaction between these two proteins is necessary. Such an interaction has been inferred for the *A. vinelandii* and *P. aeruginosa* NasS-NasT systems, but to our knowledge, no experimental evidence has yet been presented. In this work, *in vitro* experiments revealed that the NasS and NasT proteins from *P. denitrificans* co-purify as a stable heterotetrameric complex. This complex comprises the NasS and NasT proteins in a 1:1 ratio and persists during a wide range of purification methods, including affinity, strong anion-exchange, and size-exclusion chromatography. This robust NasS-NasT protein-protein interaction is, however, sensitive to NO_3_^−^ and NO_2_^−^ but not other anions.

The relative affinity of the NasS-NasT regulatory complex for NO_3_^−^ was found to be in the low micromolar range (*K*_*D*_^app^ ∼ 15 μm), which is in good agreement with the *K_m_* value of ∼17 μm reported for the NasC NO_3_^−^ reductase from *P. denitrificans* (5). Notably, this value is also consistent with that (∼5 μm) reported by Chai and Stewart (12) for the NasR-*nasF* leader RNA complex with NO_3_^−^ . In contrast, the *K*_*D*_^app^ value of NasS-NasT for NO_2_^−^ (∼94 μm) was an order of magnitude higher than the *K_m_* value of ∼5 μm reported for the NasB NO_2_^−^ reductase (5) and thus may reflect the inability of NasS to discriminate between NO_3_^−^ and the smaller chemically similar NO_2_^−^ anion. The ability of NasS-NasT to bind NO_2_^−^, albeit with lower affinity than NO_3_^−^, is consistent with *P. denitrificans* being able to grow with millimolar levels of NO_2_^−^ as the sole nitrogen source. Given that NO_3_^−^ and NO_2_^−^ are assimilated via a common pathway, there may be a selective advantage for some bacteria to express a sensor with dual specificity that is capable of detecting the presence of both inorganic nitrogen sources.

Significantly, exposure to NO_3_^−^ or NO_2_^−^ triggered dissociation of the heterotetrameric NasS-NasT complex (apparent molecular mass of ∼134 kDa) into monomeric NasS (apparent molecular mass of ∼38 kDa) and a homo-oligomeric state of NasT (apparent molecular mass of ∼130 kDa). Given the error of the gel-filtration experiment and that NasT is a low molecular mass protein, the multimeric state was broadly consistent with a hexamer.

In *P. aeruginosa*, the AmiC and AmiR proteins act to mediate inducer-responsive regulation of the *amiEBCRS* operon, which encodes the necessary genes for catabolic degradation of aliphatic amides (30). Notably, the ANTAR protein AmiR has been structurally resolved with its cognate small molecule-binding partner AmiC in a heterotetrameric ligand-responsive regulatory complex, (AmiC-AmiR)_2_ (29). When considered with the genetic results presented for *nasS* and *nasT* strains, the biochemical properties of NasS-NasT suggest that, prior to NO_3_^−^/NO_2_^−^-dependent induction of *nas* gene expression, a ligand-free NasS-NasT complex exists in which NasT is inactive. Thus, the NasS-NasT and AmiC-AmiR regulatory systems may share mechanistic similarities.

The regulatory mechanism of NasR, whose target is a hairpin in the leader RNA of *nasF*, the promoter-proximal gene of the *nas* operon in *Klebsiella* sp., has been extensively studied (11, 15, 25). Transcription antitermination control mechanisms mediated by NasT have also been postulated to regulate *nas* gene expression (10, 28). In support, we present evidence from electrophoretic mobility shift assays that, in the presence of NO_3_^−^, NasT is able to bind the leader RNA of the *nasA* gene, which contains putative regulatory elements. Formation of this NasT-*nasA* RNA complex is consistent with the proposed regulatory interaction required for ANTAR-type signaling proteins.

**FIGURE 8.**
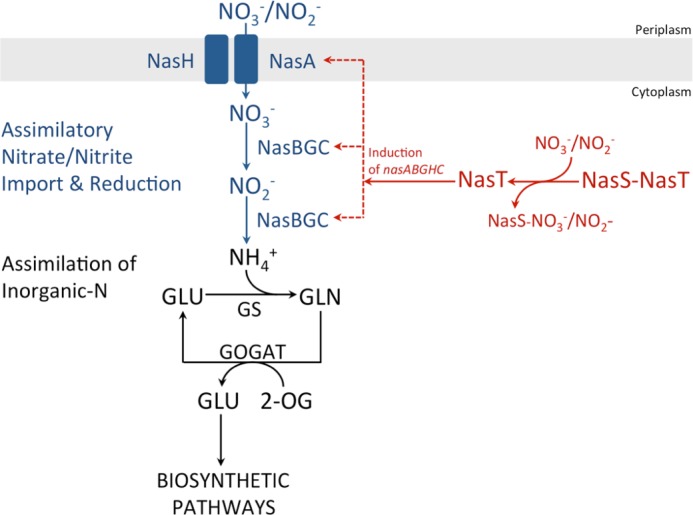
**Model for NO_3_^−^/NO_2_^−^-dependent induction of the NO_3_^−^ assimilation pathway in *P. denitrificans* mediated by NasS-NasT.** *GLU*, l-glutamate; *GLN*, l-glutamine; *GS*, glutamine synthetase; *GOGAT*, glutamine:2-oxoglutarate amidotransferase; *2-OG*, 2-oxoglutarate.

The data presented herein for NasS-NasT suggest that, following inducer perception by this NO_3_^−^/NO_2_^−^-responsive regulatory complex, the ANTAR-type protein NasT is released from the complex with NasS. Once free, NasT can activate transcription of the *P. denitrificans nasABGHC* gene cluster necessary for the reductive assimilation of this nitrogen source (Fig. 8).

Finally, the structural basis of the protein-RNA interaction remains poorly understood, but an oligomeric form of an ANTAR-type protein, as suggested here for NasT, may be functionally relevant. In this respect, it is notable that AmiR has also been shown to form oligomers of a similar size range after inducer-mediated dissociation of the (AmiC-AmiR)_2_ regulatory complex (29) and that other recognized RNA-binding proteins such as Hfq are functional as homohexamers (31).

## Footnotes

4 The abbreviations used are:

NAS: assimilatory nitrate/nitrite reductase system
IMAC: immobilized metal affinity chromatography.
